# Life cycle assessment of a LiFePO_4_ cylindrical battery

**DOI:** 10.1007/s11356-024-32543-3

**Published:** 2024-03-01

**Authors:** Manuel Botejara-Antúnez, Alejandro Prieto-Fernández, Jaime González-Domínguez, Gonzalo Sánchez-Barroso, Justo García-Sanz-Calcedo

**Affiliations:** Departamento de Expresión Gráfica, Universidad de Extremadura, Avenida de Elvas, s/n, Badajoz, 06006 Spain

**Keywords:** Life cycle assessment, Lithium-ion batteries, LiFePO_4_ battery, Environmental impacts, Eco-design

## Abstract

Reduction of the environmental impact, energy efficiency and optimization of material resources are basic aspects in the design and sizing of a battery. The objective of this study was to identify and characterize the environmental impact associated with the life cycle of a 7.47 Wh 18,650 cylindrical single-cell LiFePO_4_ battery. Life cycle assessment (LCA), the SimaPro 9.1 software package, the Ecoinvent 3.5 database and the ReCiPe 2016 impact assessment method were used for this purpose. Environmental impacts were modelled and quantified using the dual midpoint-endpoint approach and the “cradle-to-gate” model. The results showed the electrodes to be the battery components with the highest environmental impact (41.36% of the total), with the negative electrode being the most unfavourable (29.8 mPt). The ageing, calibration and testing process (53.97 mPt) accounts for 97.21% of the total impact associated with the production process’s consumption of energy, and 41.20% of the total impact associated with the battery. This new knowledge will allow a more detailed view of the environmental impact of cylindrical cell LiFePO_4_ batteries, favouring the identification of critical points to enhance their sustainable production.

## Introduction

Electric batteries are one of the alternative electrochemical energy storage for the decarbonization of the energy sector (Salama et al. 2023). One type is that of lithium-ion (Li-ion) batteries. These have remarkable properties including high specific power, efficient gravimetric and volumetric energy densities, small size, good storage capacity, low self-discharge rate and a lack of memory effect (Arshad et al. 2020). Among the most used Li-ion batteries are those based on lithium cobalt oxide (LiCoO_2_), lithium manganese oxide (LiMn_2_O_4_), lithium, nickel, manganese and cobalt oxide (Li[Ni_x_Co_y_Mn_z_]O_2_), and lithium iron phosphate (LiFePO_4_) (Mahmud et al. 2022). This last is the Li-ion type with the longest and more durable life cycles (Li et al. 2018).

Another parameter to take into account in the design of a Li-ion battery is the type of cell. For the LiFePO_4_ type, there are different cell formats: cylindrical, button, prismatic and pouch (Murashko 2016). The choice of format is generally associated with the needs of the use and/or destination of the battery, with the cylindrical type being the commonest principally because of its great mechanical stability and ease of production (Yuan et al. 2016). In addition, this cell is equipped with a pressure relief valve that prevents any internal anomaly, thus protecting it from any possible deformations due to overpressure (Li et al. 2021).

There are different standardized sizes of Li-ion cylindrical cells (14,240, 15,266, 16,340, 18,650, 21,700, 22,430, 26,650, etc.), although there are no national or international standards regulating them (Korthauer 2018; Li 2022). The 18,650 format corresponds to an 18-mm diameter, 65-mm-long cylindrical cell (Quinn et al. 2018). It was first launched as a standard Li-ion battery model in 1994 and has since then become one of the most used (Xu et al. 2021), for instance, in electronic cigarettes (Saxena et al. 2018), powerbanks (Diao et al. 2020), electric vehicles (Uitz et al. 2017) and even orbital nanosatellites (CubeSats) (Krause et al. 2020). Currently, annual increases of 1.7% in production volume are anticipated (Xu et al. 2021), translating into a considerable rise in demand for the materials required in their manufacture (Peters et al. 2017).

The materials used in the different components that make up the batteries and the corresponding production methods are ultimately accountable for the environmental impacts associated with the life cycle of a battery (Wang et al. 2019). For this reason, it is becoming increasingly necessary to analyse and categorize the impact associated with each of the components and processes involved (Lai et al. 2022a) in order to study new more sustainable solutions and/or alternative production processes that can reduce the environmental impact associated with the battery production (Zubi et al. 2018).

Energy and environmental problems have become ever more important in recent years (Qadeer et al. 2023). This has generated great social concern which has been transferred to the field of science and has led to the development of a bibliography focused on the study of the environmental impact associated with the life cycle of the different types of batteries (Arshad et al. 2022; Zhou et al. 2023). In the field of Li-ion batteries, various researchers explored the impact associated with the life cycle.

Dai et al. (2019) analysed the environmental impact associated with the production of a 1-kWh-cylindrical cell Li-ion NMC111 (LiNi_1/3_Mn_1/3_Co_1/3_O_2_) battery. For this purpose, they adopted a “cradle-to-gate” approach and used GREET® software (Wang et al. 2022). The research mainly focused on greenhouse gas (GHG) emissions (So_x_, NO_x_ and PM_10_), not considering other significant environmental impact categories such as IR, HCT and TA. Furthermore, this software does not consider the endpoint impact analysis, not allowing to group the impact of the solutions studied by protection area. Likewise, Marques et al. (2019) carried out a comparative LCA of 24-kWh LiMn_2_O_4_ and LiFePO_4_ cylindrical cell battery typologies. In their study, they used a “cradle-to-grave” approach and the CML-IA impact assessment method (Van Oers 2015). This method does not allow impact analysis by protection areas. ReCiPe is an updated version of that method and has been significantly improved to provide segmented impact by protection area, which is not possible under the CML-IA method.

Liang et al. (2017) considered a 1000-kWh LiFePO_4_ single-cell button cell battery as a functional unit and calculated its carbon footprint, which is limited in scope compared to a comprehensive LCA. Hence, disregarding the remaining ones, they only analysed five of the 17 midpoint categories of the ReCiPe method. In their critical literature review, Lai et al. (2022a, b) disaggregated the scope of LCA on Li-ion batteries, the types of studies and the future challenges of this topic. In that work, they found that LCAs have focused on carbon footprint calculations. Lai et al. (2023) continued their line of research by comparing the LCA of five Li-ion and six Na-ion battery types. Specifically, they focused on the manufacturing process (“cradle-to-gate”) for a 1-kWh functional unit, based on different potentials (global warming potential, acidification potential, etc.) (Heijungs 2014), and grouped into the impact categories global warming (GW), acidification (AF), human toxicity (HT), land use (LU) and metal and minerals (MM) through Gabi software inventory flows (Herrmann and Moltesen 2015). Therefore, they applied a customised impact assessment method, which is not widely used in the scientific community and may pose an issue for comparing these results (Rosenbaum et al. 2018). In addition, it should be noted that they did not consider the endpoint perspective in their analysis process. Kim et al. (2023) analysed the carbon footprint of a 1-kWh Li-ion battery using an impact assessment method that is not widely used in the scientific literature (IPCC’s Fifth Assessment Report (AR5)). To this end, they considered a “cradle-to-gate” approach adding the use phase. However, like the other works, they focused on the GW category without considering the other impact categories and protection areas.

Other authors have carried out LCA on Li-ion batteries but without comprehensively analysing all midpoint impact categories and endpoint protection areas. Thus, Ambrose and Kendall (2016) analysed the environmental impact of different recycling strategies for the 1-ton-functional unit of cylindrical cell Li-ion batteries. Specifically, they focused their analysis on the GHG emission calculation, disregarding other relevant midpoint categories (such as HCT, SODP, ME and LU) and omitting the analysis of impacts by protection area endpoint. Then, Sadhukhan and Christensen (2021) calculated the environmental impact of a prismatic cell Li-ion battery (they did not specify its power) from a “cradle-to-cradle” approach and using different impact assessment methods (ReCiPe, CML and ILCD). However, they focused their study on the midpoint GW category, not analysing the rest of the impact categories or the endpoint approach. Fan et al. (2023) compared the LCA of four Li-ion battery types based on LiFePO_4_ (LFP) and lithium nickel cobalt manganese oxide (NCM) typologies. In their research, they adopted a “cradle-to-cradle” approach, using the CML-IA impact assessment method for a functional unit of 1 kWh. Therefore, they disregarded the analysis of aggregate environmental impacts by protection area endpoint. Chen and Hsieh (2023) carried out an analysis of the carbon footprint associated with different Li-ion battery recycling strategies based on the IPCC’s Sixth Assessment Report (AR6) method, which only considers the GW impact category. For this purpose, they evaluated six battery types — one LFP, one lithium nickel cobalt aluminium oxide (NCA) and four NCM (NCM111, NCM532, NCM622 and NCM811) — of 1 kWh. Like all the other authors mentioned above, they focused their research on GHG emissions.

Recently, two lines of work have emerged to act on the environmental impact of Li-ion batteries. On the one hand, modifying the design process of Li-ion batteries was proposed by Akasapu and Hehenberger (2023), based on the information available in the scientific literature. However, their proposal was based on the most widely used protection categories in the literature, which were GW and abiotic depletion (ADP). On the other hand, assessing the LCA of different recycling strategies as the following authors, Islam and Iyer-Raniga (2022), they conducted a state-of-the-art review of Li-ion batteries from the point of view of recyclability and circular economy. They focused on the analysis of techniques and study topics. Then, Goyal et al. (2023) updated the previous literature review on this topic. Finally, Liu et al. (2023) evaluated the life cycle of different recycling scenarios for LiFePO_4_ and NCM (ternary lithium) 57-kWh Li-ion batteries using GaBi software and a customized impact assessment method (Pauer et al. 2020).

In summary, most of the analyses carried out in the state of the art focused on impact categories related to the carbon footprint, mainly on the GW and AF categories. Consequently, it seems that there is a need for research that analyses all 22 impact categories and the three protection areas using the ReCiPe method, which is the most widely used, most global in scope and most recognised in the scientific community This will be of great interest to the scientific community and industry, as it allows classifying, weighting and characterising impact categories, properly interpreting their influence on environmental impacts in such interesting areas as human health.

### Research novelty and objectives

The novelty of the present investigation is the inclusion of the environmental dimension in the life cycle of cylindrical cell LiFePO4 batteries, specifically those of the 18,650 formats, where the casing was considered for which the LCA method was employed (Porzio and Scown 2021). LCA is a tool for environmental management and analysis used to quantify the wide range of potential environmental consequences of a product or system throughout its life cycle (from the extraction of the raw materials to the disposal phase, taking into account the stages of production and use of the product, process or system analysed) (Wu and Kong 2018; Sánchez-Barroso et al. 2021). It also helps in estimating ecosystem quality and the impact on human health and thus informs decision-makers in industry and governmental or non-governmental organizations of this matter (Jiang et al. 2020; Botejara-Antúnez et al. 2022). In addition, LCA is used to improve the environmental efficiency of the battery manufacturing process, introducing a novel and significant variable into the decision-making process (Wang et al. 2020).

The main objective of this research was to model, determine and quantify the environmental impact associated with the stages of extracting the raw materials and producing a 7.47-Wh 18,650 cylindrical cell LiFePO_4_ battery. If the current *status quo* continues, Degen (2023) estimated that approximately 5.86 Mt CO_2_ eq will be emitted by 2030 due to the energy demand of European Li-ion battery cell production, which could be reduced by 46–56% by applying a combination of mainly technological measures. In this way, this work will allow to quantify the environmental impact associated with the whole battery and with each of its components and production processes, identifying the main sources of environmental penalization and favouring the proposal of more sustainable alternative productive models (eco-design) aligned with the UN’s Sustainable Development Goals (United Nations 2015).

## Material and methods

### General method

The LCA method was used for the analysis and assessment of the environmental impacts associated with the life cycle of the cylindrical cell Li-ion battery. Its procedures are based on the regulatory framework of ISO 14040 (International Organization for Standardization 2020) and ISO 14044 (International Organization for Standardization 2017). The SimaPro 9.1 software package was employed to model the battery stack with the objective of assessing the different elements comprising it and quantifying its characteristic environmental performance (PRé Sustainability B.V. 2020). The Ecoinvent 3.5 database was chosen because it offers a full range of life cycle inventories (LCI) and allows the use of various impact assessment methods (Ecoinvent Association 2018). The impact assessment method selected was ReCiPe 2016 of recognized international prestige and characterized by its dual (midpoint and endpoint) approach (Huijbregts et al. 2017).

### System boundaries and functional unit

The study consisted of a “cradle-to-gate” assessment of a cylindrical cell Li-ion battery and its related production processes, including the stages between the extraction of raw materials and the production of the Li-ion battery under study up to the factory gate, before it is distributed to customers. This approach was chosen since it allows the identification of calls to action in order to reduce the environmental impact of its production, regardless of the particular use of the battery. In addition, it is the appropriate approach to state the Environmental Product Declaration. To carry out the LCA, it was necessary to establish a reference unit with the objective of appropriately relating the inputs and outputs of the production process of the cylindrical cell Li-ion battery and its components and characteristic parameters. The functional unit (FU) established was a 7.47-Wh 18,650 cylindrical single-cell LiFePO_4_ battery unit. Also included was a sensitivity analysis of the impacts of battery production per kilometre distance in those phases of raw material extraction and component production and assembly. This sensitivity analysis assessed how much the environmental impact of the battery is influenced by the distances associated with the supply of raw materials and casings and the location of the production point.

### Impact categories

The ReCiPe 2016 method was applied, as it is one of the most used approaches in impact assessments (Huijbregts et al. 2016). It is characterized by expressing the environmental impact results from a twofold perspective (midpoint and endpoint) which are organized into a series of individually parameterized impact categories. Concretely, for endpoint perspective, the impact categories are also called “protection areas”. Table 1 lists these impact categories classified by environmental assessment perspective: midpoint and endpoint.

**Table 1:** Impact categories employed in the life cycle assessment under ReCiPe 2016 method

Perspective	Impact category	Acronym	Units	Impact category	Acronym	Units
Midpoint	Global warming, human health	GWHH	kg CO_2_ eq	Terrestrial acidification	TA	kg SO_2_ eq
	Stratospheric ozone depletion	SODP	kg CFC_11_ eq	Freshwater eutrophication	FE	kg P eq
	Ionizing radiation	IR	kBq Co-60 eq	Marine eutrophication	ME	kg N eq
	Ozone formation, human health	OFHH	kg NO_x_ eq	Terrestrial ecotoxicity	TE	kg 1,4-DCB
	Fine particulate matter formation	FPMF	kg PM_2.5_ eq	Freshwater ecotoxicity	FEC	kg 1,4-DCB
	Human carcinogenic toxicity	HCT	kg 1,4-DCB	Marine ecotoxicity	MEC	kg 1,4-DCB
	Human non-carcinogenic toxicity	HnCT	kg 1,4-DCB	Land use	LU	m^2^a crop eq
	Water consumption, human health	WCHH	m^3^	Water consumption and terrestrial ecosystems	WCTE	m^3^
	Global warming, terrestrial ecosystems	GWTE	kg CO_2_ eq	Water consumption and aquatic ecosystems	WCAE	m^3^
	Global warming, freshwater ecosystems	GWFE	kg CO_2_ eq	Mineral resource scarcity	MRS	kg Cu eq
	Ozone formation, terrestrial ecosystem	OFTE	kg NO_x_ eq	Fossil resource scarcity	FRS	kg oil eq
Endpoint	Human health	HH	DALY			
	Ecosystems quality	EQ	species·yr			
	Resource availability	RA	US$			

### Description case

This study analysed an 18,650 cylindrical cell Li-ion battery. The first four digits of this battery refer to the physical dimensions — 18 mm in diameter and 65 mm in height, and the latest digit 0 indicates the cylindrical cell format. This type of battery is characterized by high energy storage capacity, high resistance to discharge and low maintainability. Each 18,650 unit is manufactured with a cathode (electrode +) based on LiFePO_4_ and an anode (electrode −) based on graphite. The battery weighs 47 g, of which only 20% corresponds to the casing. Its energy capacity is 7.47 Wh and, under normal use condition, the efficiency ranges from 85 to 98%. Finally, given that the useful life of a battery is the number of cycles that it can sustain before its nominal capacity falls below 80%, this battery is expected to achieve a nominal 500 cycles for a depth of discharge (DoD) that is stable over time. Table 2 lists in detail the full technical specifications of the 18,650 cylindrical cell Li-ion battery tested.

**Table 2:** Technical specifications of 18,650 cylindrical cell LiFePO_4_ battery

	Technical specifications
Nominal capacity	2600 mAh (0.2 C, 2.75 V discharge)
Min. capacity	2550 mAh (0.2 C, 2.75 V discharge)
Charging voltage	4.2 ± 0.05 V
Nominal voltage	3.7 V
Charging method	DC-CV (constant voltage with current limit)
Charging current	Standard charging 1300 mA Fast charging 2600 mA
Charging time	Standard charging 3 h Fast charging 2.5 h
Max. charging current	2600 mA (ambient temperature 25 °C)
Max. discharge current	5200 mA (ambient temperature 25 °C)
Cut-off discharge voltage	2.75 V
Battery weight	47 g
Cell dimensions	Height 65 mm max
Diameter 18.40 mm max	
Operating temperature	Charge 0–45 °C
Discharge − 20–60 °C	
Storage temperature	1 year − 20 ~ 25 °C
3 months − 20 ~ 45 °C	
1 month − 20 ~ 60 °C	

**Fig. 1:**
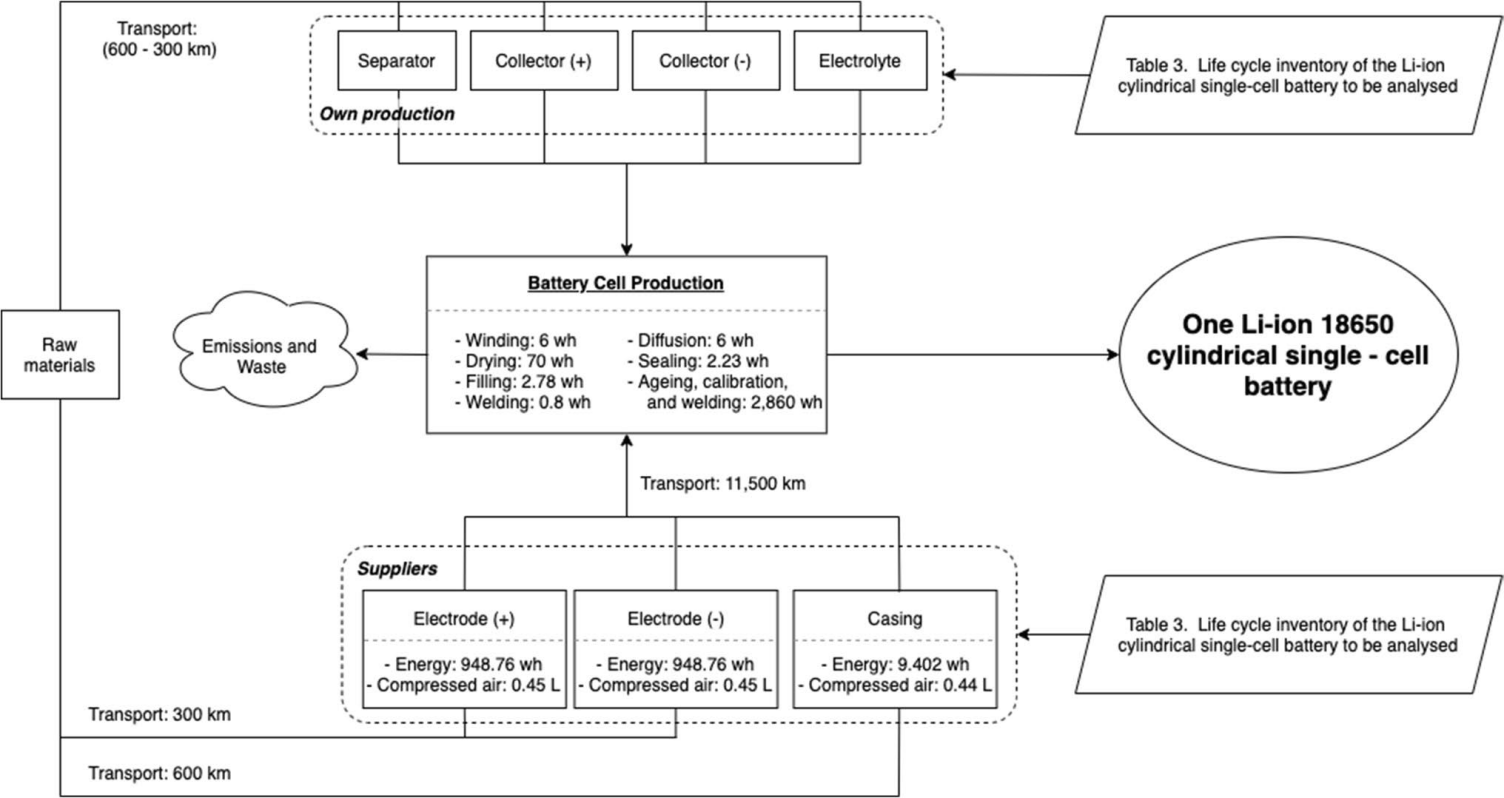
LiFePO_4_ 18,650 battery simplified flow diagram

It was opted to take a mixed production cycle for the manufacture of the Li-ion battery detailed in Table 2, combining self-manufacture in a factory in Madrid (Spain) with the acquisition of some components — electrodes and casing — from suppliers located in Beijing (China). Figure 1 illustrates the itinerary and life cycle flow followed to produce the 18,650 cylindrical cell LiFePO_4_ battery functional unit.

### Life cycle inventory

In this study, the life cycle inventory (LCI) data are derived from surveys of companies from the sector, open-source data from relevant scientific literature, industry statistical yearbooks and government regulations and standards. In addition, background data are based on SimaPro 9.1 software (PRé Sustainability B.V. 2020) and the Ecoinvent 3.5 database (Ecoinvent Association 2018).

Moreover, based on the selected “cradle-to-gate” approach, the LCI of the battery included data on raw material acquisition, component manufacturing, all materials used in the battery assembly, as well as energy, emissions, and waste. For emissions and waste, pre-established values from the Ecoinvent 3.5 database was considered (Hauschild and Bjørn 2018). Furthermore, this study broke down the Li-ion battery into several parts, such as the separator, the electrode ( +), the electrode ( −), the collector ( +), the collector ( −), the electrolyte and the casing. The main material of the anode (electrode −) is graphite, while the cathode (electrode +) material is LiFePO_4_, which is synthesized from polyvinylidene fluoride (PVDF) and carbon black. Table 3 lists the LCI of the battery whose flow chart is presented in Fig. 1.

**Table 3:** Life cycle inventory of the LiFePO_4_ cylindrical single-cell battery to be analysed

Components	Materials	Weight (kg)	Transport (km)	
			Mine to battery factory / supplier factory	Supplier factory to battery factory
Separator (#1)	Polypropylene	4.34·10^−4^	600	-
Electrode ( +) (#2)	Carbon black	1.20·10^−3^	300	11,500
	LiFePO_4_	6.51·10^−3^	350	
	PVDF	3.20·10^−4^	600	
Electrode ( −) (#3)	Carbon black	6.00·10^−4^	300	11,500
	Graphite	3.25·10^−3^	300	
	PVDF	1.60·10^−4^	600	
Collector ( +) (#4)	Aluminium	1.45·10^−3^	600	-
Collector ( −) layer (#5)	Copper	3.60·10^−3^	600	-
Electrolyte (#6)	Ethylene carbonate	1.07·10^−3^	300	-
	Dimethyl carbonate	1.07·10^−3^	300	
	LIPF_6_	2.15·10^−3^	600	
Casing (#7)	Nickel-plated steel	9.5·10^−3^	600	11,500

Furthermore, Table 4 lists the energy consumption associated with the production process of the Li-ion cylindrical single-cell battery, where the contribution of the electrodes, and the casing production process has also been considered. The consumption of the “Ageing, calibration and testing” process — P7 — is so high compared to the rest because each FU is tested individually, while the other six processes take advantage of economies of scale to minimise the energy impact associated with the FU.

**Table 4:** Consumptions associated with the battery production process

	Winding (P1)	Drying (P2)	Filling (P3)	Welding (P4)	Diffusion (P5*)*	Sealing (P6)	Ageing, calibration and testing (P7)	Total
Electricity consumption (kWh)	6.00·10^−6^	0.07	2.78·10^−3^	8.00·10^−4^	6.00·10^−3^	2.23·10^−3^	2.86	2.94

### General results

Table 5 gives the LCA results by impact category for each of the components of the cylindrical cell Li-ion battery and the different types of consumption associated with its production process.

**Table 5:** Life cycle assessment results by impact category

Impact categories	Impact associated with the battery components							Consumptions in the battery production process	Total
	Separator	Electrode ( +)	Electrode ( −)	Collector ( +)	Collector ( −)	Electrolyte	Casing		
*GW_T_ (kg CO_2_ eq)	2.1·10^−3^	0.46	0.44	4.3·10^–3^	0.04	0.05	0.07	1.00	2.10
SODP (kg CFC_11_ eq)	2.2·10^−11^	2.4·10^−7^	2.5·10^−7^	2.6·10^−9^	6.4·10^−8^	1.8·10^−8^	2.4·10^−8^	5.5·10^−7^	1.2·10^−6^
IR (kBq Co-60 eq)	9.9·10^−7^	0.22	0.22	2.5·10^−4^	8.1·10^−3^	4.6·10^−3^	4.4·10^−3^	0.58	1.00
OFHH (kg NO_x_ eq)	4.1·10^−6^	1.5·10^−3^	1.5·10^−3^	1.4·10^−5^	2.2·10^−4^	1.1·10^−4^	2.3·10^−4^	3.6·10^−3^	7.1·10^−3^
FPMF (kg PM2.5 eq)	1.9·10^−6^	1.1·10^−3^	1.2·10^−3^	1.1·10^−5^	4.1·10^−4^	1.1·10^−4^	2.0·10^−4^	2.7·10^−3^	5.8·10^−3^
OFTE (kg NOx eq)	4.1·10^−6^	1.5·10^−3^	1.5·10^−3^	1.5·10^−5^	2.2·10^−4^	1.1·10^−4^	2.3·10^−4^	3.6·10^−3^	7.1·10^−3^
TA (kg SO_2_ eq)	6.4·10^−6^	2.7·10^−3^	3.1·10^−3^	2.6·10^−5^	1.2·10^−3^	2.9·10^−4^	2.8·10^−4^	6.8·10^−3^	0.01
FE (kg P eq)	4.6·10^−8^	2.4·10^−4^	3.7·10^−4^	4.2·10^−6^	3.0·10^−4^	2.0·10^−5^	2.2·10^−5^	5.0·10^−4^	1.5·10^−3^
ME (kg N eq)	4.4·10^−9^	2.2·10^−5^	2.7·10^−5^	3.7·10^−7^	1.8·10^−5^	5.8·10^−6^	1.4·10^−6^	4.2·10^−5^	1.2·10^−4^
TE (kg 1,4-DCB)	6.6·10^−4^	1.30	4.80	0.05	7.50	0.20	1.10	2.60	17.5
FEC (kg 1,4-DCB)	7.3·10^−7^	0.04	0.06	1.1·10^−3^	0.05	1.6·10^−3^	3.3·10^−3^	0.10	0.25
MEC (kg 1,4-DCB)	1.3·10^−6^	0.04	0.08	1.4·10^−3^	0.08	2.3·10^−3^	4.8·10^−3^	0.12	0.33
HCT (kg 1,4-DCB)	1.1·10^−6^	0.03	0.03	6.4·10^−4^	0.01	1.9·10^−3^	0.02	0.05	0.15
HnCT (kg 1,4-DCB)	2.9·10^−5^	0.37	1.30	0.02	2.00	0.05	0.06	0.85	4.60
LU (m^2^a crop eq)	2.1·10^−6^	0.01	0.02	1.5·10^−4^	3.3·10^−3^	1.4·10^−3^	2.3·10^−3^	0.03	0.07
MRS (kg Cu eq)	1.8·10^−7^	4.6·10^−3^	3.2·10^−3^	2.2·10^−4^	4.3·10^−3^	1.5·10^−3^	4.8·10^−3^	2.8·10^−3^	0.02
FRS (kg oil eq)	1.0·10^−3^	0.13	0.12	1.1·10^−3^	0.01	0.01	0.02	0.28	0.58
**WC_T_ (m^3^)	2.1·10^−5^	0.03	0.03	1.3·10^−4^	5.3·10^−4^	9.3·10^−4^	3.8·10^−4^	0.01	0.07

*GWT = GWHH + GWTE + GWFE

**WCT = WCHH + WCTE + WCAE

One observes that the main impacts of the Li-ion battery analysed come from the consumption associated with the cell battery’s production process (CBPP), with maximum scores in 14 out of the 18 impact categories, and with values between 0.15 and 1.26 times higher than the impact associated with the set of components of the battery’s stack. These figures are similar to those reported by other authors such as Dai et al. (2019), who demonstrated how the environmental impact associated with the energy flows of the production process of a 1-kWh NCM111 Li-ion battery led the majority of the impact categories — 71.42% of the total — considered in their carbon footprint study.

Table 6 provides a compilation of some of the LCA studies carried out in the field of Li-ion batteries in recent years and their main results.

**Table 6:** Compilation of LiFePO_4_ battery publications and their main results

Researcher	Functional unit	Life Cycle Impact Assessment method	Battery type	Main results	
				GW (kg CO_2_ eq)	AF (kg SO_2_ eq)
Liang et al. (2017)	1000 kWh	EPD	LiFePO_4_	720.70	-
Marques et al. (2019)	1 kWh	CML-IA	LiFePO_4_	7713.00	69.70
Salgado Delgado et al. (2019)	10 kWh	ReCiPe 2008	LiFePO_4_	0.27 kg CO_2_ eq/Wh	-
Lai et al. (2023)	1 kWh	GaBi	LiFePO_4_	62.00 CO_2_ eq/kg	-
Fan et al. (2023)	1 kWh	CML-IA	LiFePO_4_	78.00 kg CO_2_ eq/kg	0.04 kg SO_2_ eq/kg

Comparing the results of the present study with those obtained by other authors who carried out an LCA of the same type of battery — LiFePO4 cylindrical cell, a certain similarity can be appreciated. For example, Marques et al. (2019) obtained a global warming (GW) of 7 713.0 kg CO_2_ eq and an acidification (AF) of 69.7 kg SO_2_ eq for a 24 kWh FU, which is 3212.85 times our 7.47 Wh FU. In rescaling, that study would have resulted in an impact of 2.4 kg CO_2_ eq and 0.02 kg SO_2_ eq for the FU = 7.47 Wh, respectively. These slight variations are due to the use of different impact assessment methods — CML-IA vs. ReCiPe — and the variability of the environmental database versions — Ecoinvent 2.2 vs. Ecoinvent 3.5. In this line, Salgado Delgado et al. (2019) found similar trends for the 10-kWh cylindrical LiFePO_4_ cell battery in the ReCiPe 2008 method. Thus, they obtained values of 0.27 kg CO2 eq/Wh in the GW category, which, translated to the functional unit of the present study (7.47 Wh), means an impact of 2.0 kg CO_2_ eq. In this case, the slight variations observed are due to the variability of versions of the impact assessment method — ReCiPe 2008 vs. ReCiPe 2016 — and of the environmental database — Ecoinvent 3.2 vs. Ecoinvent 3.5.

Nevertheless, substantial differences were observed for other cell types. Liang et al. (2017) calculated the carbon footprint of a 1000-kWh LiFePO_4_ button cell battery, obtaining values of 720.7 kg CO_2_ eq from the Environmental Product Declaration 2008 method. These results differ from those obtained in the current study for our 7.47-Wh cylindrical LiFePO_4_ cell battery (2.1 kg CO_2_ eq). This fact is mainly due to the difference in shape and size of the main components — anode and cathode — as a reason for the different cell morphology which causes variations in the amount of CO_2_ eq needed to produce the cell. Furthermore, the characterization factors were also different due to the use of different impact assessment methods — EPD vs. ReCiPe 2016. Finally, it should be noted that the environmental database used by Liang et al. (2017) is limited, with only 300 processes, while the Ecoinvent 3.5 database has more than 12,500, which allows for a better fit in modelling the environmental profile of the battery under study (Reinhard et al. 2019).

**Fig. 2:**
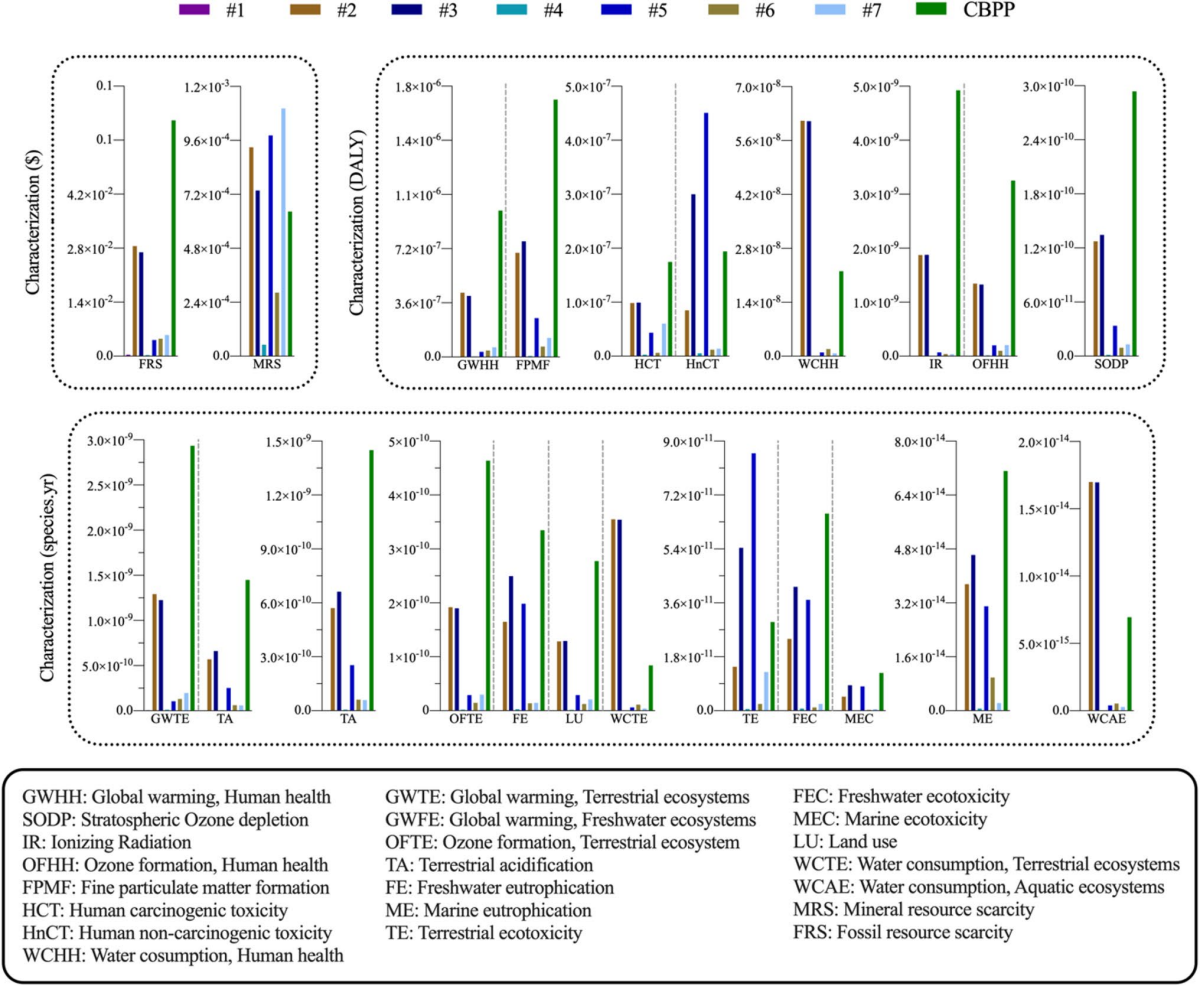
Characterization of impact categories

Figure 2 shows the results of the impact category characterization process. This quantification established the characterization of the three future protection areas —Human health, Ecosystem quality and Resource availability — based on three measurement scales (US$, DALY and species·year) and is essential to understanding the flow of damage followed in the later stages of the LCA method.

For the human health characterization, the most unfavourable score for an impact category was FPMF, in which the electrode ( +) and electrode ( −) of the Li-ion battery and the energetic contributions of the CBPP stand out, with CBPP being the source of highest impact (1.7·10^−6^ DALY). With respect to overall ecosystem quality characterization, the electrode ( +) and electrode ( −) components again stand out over the rest, only being surpassed by CBPP and generating the highest impacts in the GWTE category (1.3·10^−9^ species·year, 1.2·10^−9^ species·year and 2.93·10^−9^ species·year, respectively). Finally, for the characterization of resource scarcity characterization, the same electrode ( +) and electrode ( −) components and CBPP once again are the most harmful impact sources and scored the highest in the FRS category (US$ 0.03, US$ 0.03 and US$ 0.06, respectively). All these trends are attributable to the high presence of SO_2_ and PM_2.5_, as a direct consequence of the different transport flows associated with the battery production cycle and the energy flows of the Spanish energy mix (World Health Organization 2021), especially those related to coal and/or the combined cycle power plants (Lestari et al. 2020).

**Fig. 3:**
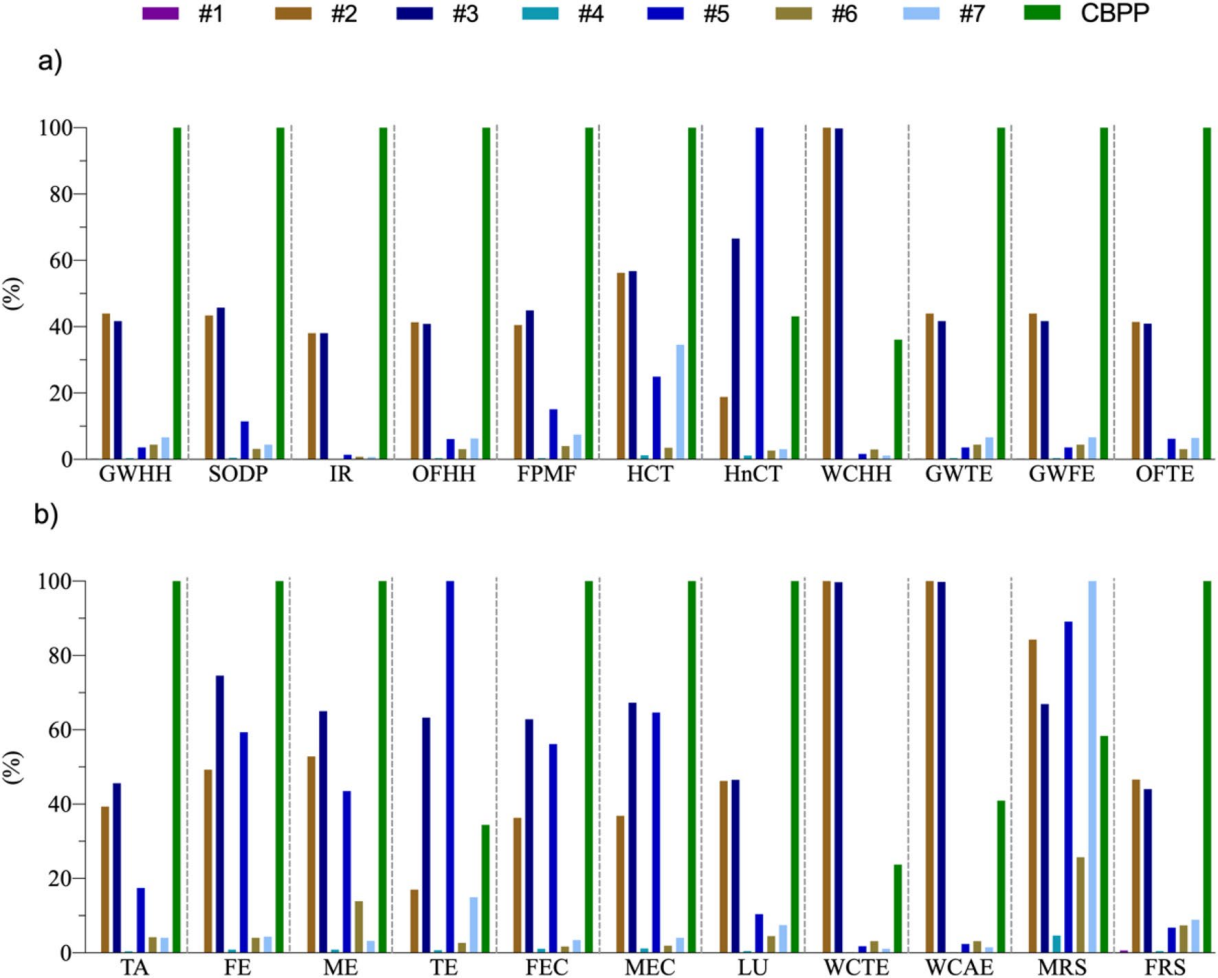
Internally standardized characterization of intermediate impact categories

Figure 3 shows the results of the internal normalization process. The value of 100% is assigned to the system with the highest score in each category, and the impact ratio of the remaining systems is set by this rescaling process.

Analysed by impact category, components electrode ( +), electrode ( −), collector ( −) and casing, and CBPP presented the highest values. Components electrode ( +) and electrode ( −) obtained the maximum values in the categories of water consumption in relation to human health (WCHH), water consumption in relation to terrestrial ecosystems (WCTE) and water consumption in relation to aquatic ecosystems (WCAE), as a consequence of the intensive water requirements related to their production process (Wood et al. 2018). The component collector ( −), obtained the highest values in the categories human non-carcinogenic toxicity (HnCT) and terrestrial ecotoxicity (TE), as a consequence of the emissions and residues derived from its manufacturing process (chromium VI, nickel, lead, etc.) (Melchor-Martínez et al. 2021; Lai et al. 2022b). The casing obtained the highest values in the mineral resource scarcity (MRS) category, and finally, the CBPP in the rest of the impact categories (16 of 22 categories), as a consequence of the great diversity of energy sources present in the Spanish energy mix (some of them are non-renewable and of great importance in the production cycle) (Gómez-Calvet et al. 2019). In general, the rest of the components presented similar values of relative importance in each impact category.

### Results according to midpoint approach

**Fig. 4:**
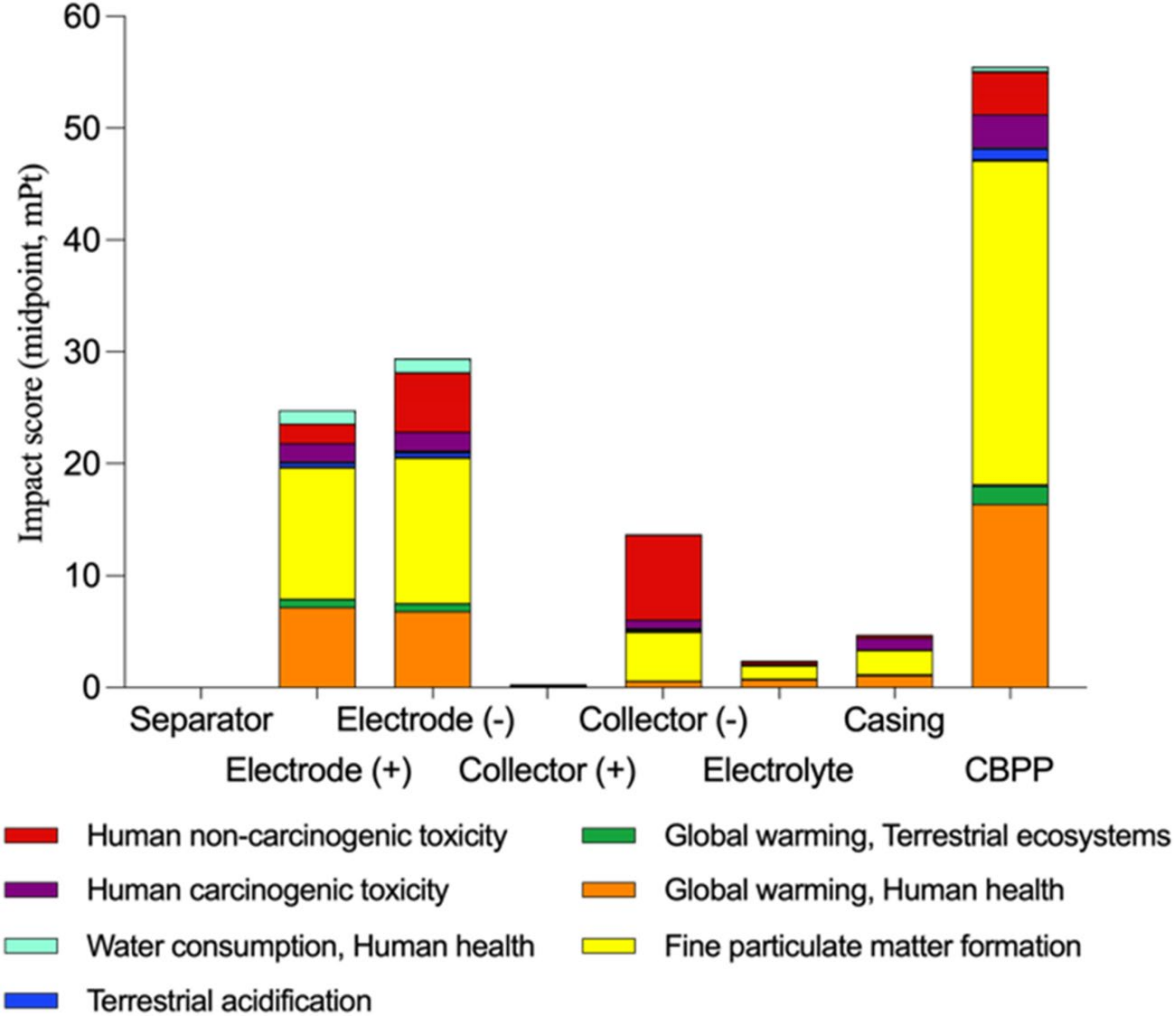
Impact analysis — unique midpoint score

Figure 4 shows the results for the individual scores of the different types of components for the midpoint categories. One can observe that the categories FPMF, WCHH and HnCT presented the highest values for the entire set of components and the Li-ion battery CBPP, with an average score of 46.74%, 25.14% and 13.64%, respectively. Thus, together, the three categories have an average score of 85.52%, which represents 91.12% of the impact associated with the Human Health endpoint area. Furthermore, among the sources of impact, the consumption associated with CBPP stands out as the most environmentally harmful (42.24% of the total impact), followed by electrode ( −) — anode — (22.44% of the total impact) and electrode ( +) — cathode — (18.74% of the total impact). Finally, it should be noted that CBPP contributions mainly penalises the FPMF (47.08%) and WCHH (49.74%) categories, while the anode has a higher impact on the HnCT category (28.28%).

The electrode ( +) and the electrode (—) present the most unfavourable environmental impact results which are between 5.82 and 6.91 times worse than the mean of the rest of the components. This fact is mainly due to their composition (Peters et al. 2017). Consequently, the main environmental penalty for the cathode comes from the LiFePO_4_ (95.06% of the impact associated to the electrode), and for the anode comes from the graphite (99.27%). This is followed by the collector ( −) and the casing, with more favourable values between 46.7 and 16.1%. Also, the collector ( −) presents values 42.46 times less favourable than the collector ( +) due to its mass (2.48 times higher) and its copper composition (Sen et al. 2019). Finally, the separator (#1) is the most favourable component with impact values 1256.67 times less than the mean of the other components. This fact is mainly due to its mass, which is 32.08 times less than the mean of the other components and its polypropylene based composition (Meshram et al. 2020).

### Results according to endpoint approach

**Fig. 5:**
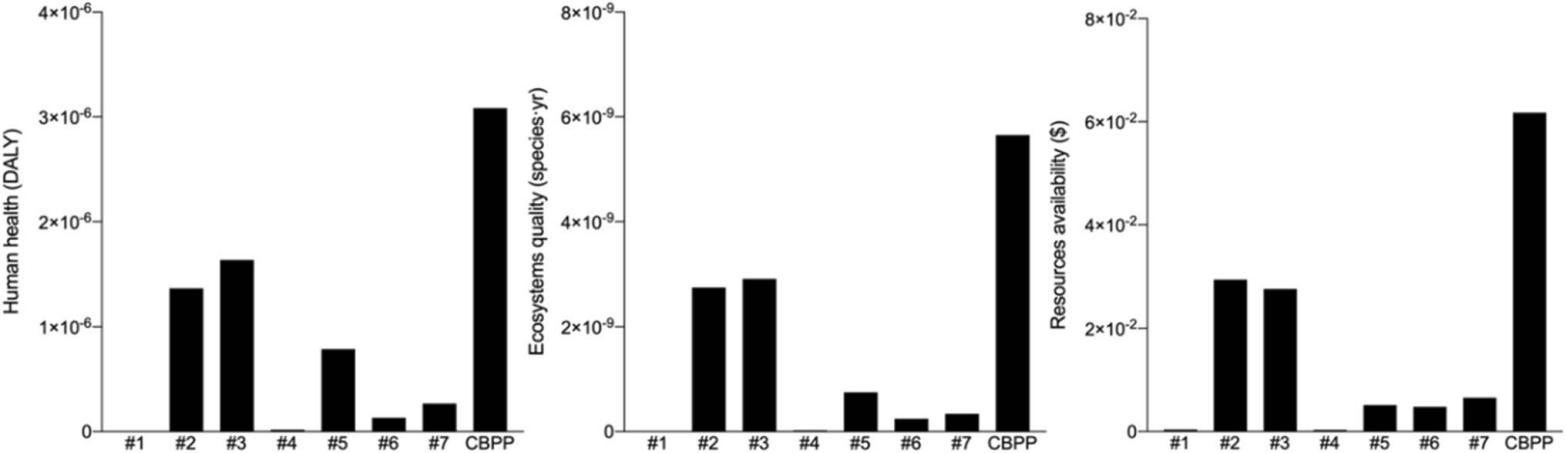
Damage assessment by protection areas

Figure 5 shows the damage assessment by protection area as an intermediate step to obtaining the single endpoint approach score. Thus, a first outline of particularized trends can be observed for each protection area, which will be consolidated in the next step from the optional LCA steps “normalisation” and “weighting”.

**Fig. 6:**
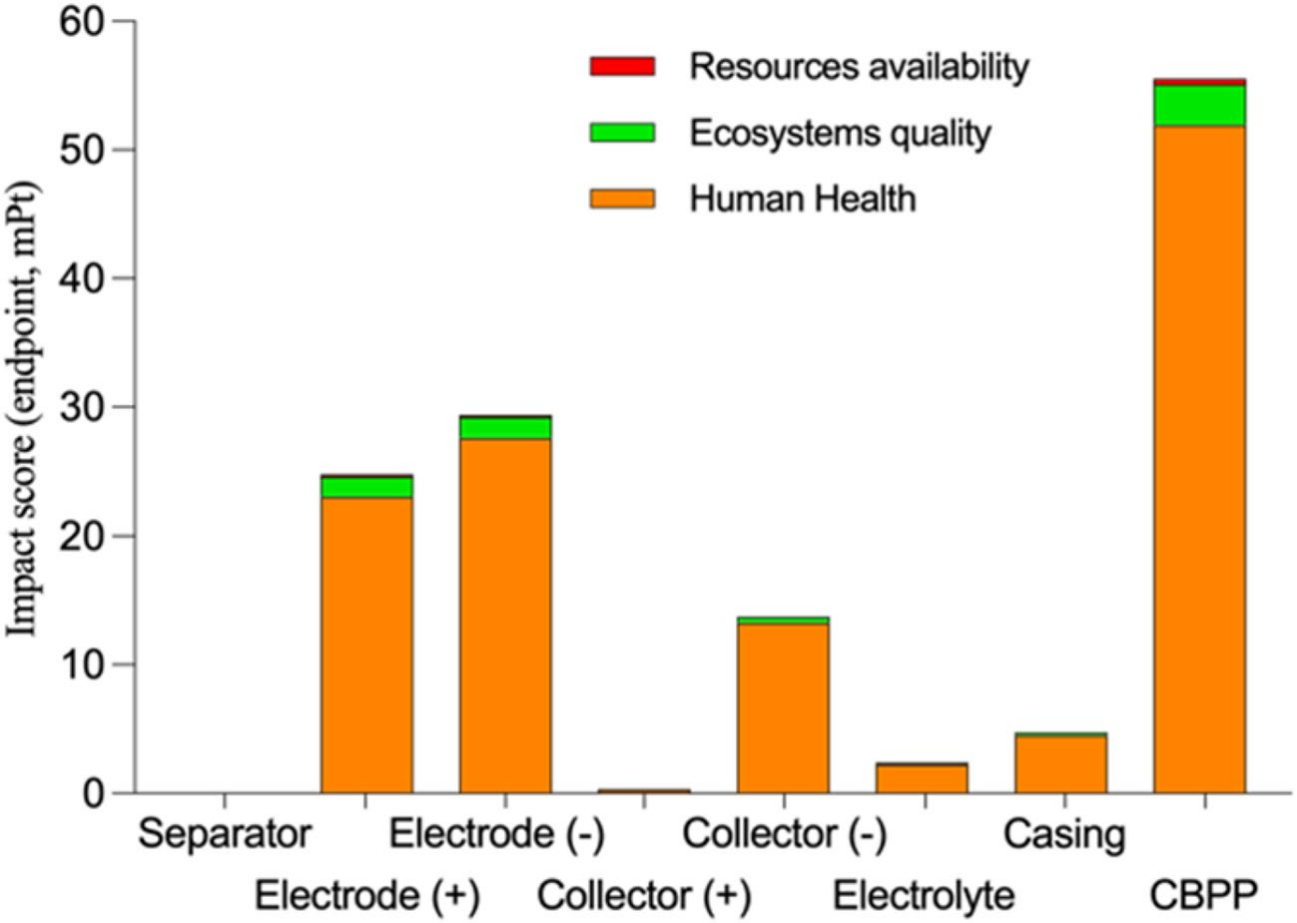
Impact analysis — unique endpoint score

Figure 6 shows the endpoint approach results for each component and the CBPP after normalizing and weighting the characterization factors. In this way, it is possible to compare the different impact sources of the Li-ion battery by aggregating the impacts in the protection areas.

Analysing the endpoint approach results for the Human Health, Ecosystem Quality and Resource Availability protection areas, one can discuss that the characteristic environmental impacts of the components of the 18,650 LiFePO_4_ cylindrical cell battery are mainly associated with the Human Health area (Sobianowska-Turek et al. 2021). This is the trend for all the components, with the mean contribution being 93.84% and influenced significantly by the impact categories FPMF, WCHH and HnCT, whose main source of impact comes from the CBPP consumption and the anode and cathode components (see Fig. 4). Together, these three categories present a mean of 85.52%, representing 91.12% of the impact associated with the final human health scoring criterion. Therefore, in line with the results obtained through the midpoint approach, it can be intuited that the environmental values of the Spanish energy matrix and the carbon present in the electrodes will be the main agents of penalization in the area of Human Health and, consequently, in the entire life cycle of the battery analysed. For the Ecosystem Quality area, the mean contribution is lower (5.41%). Finally, the impact of the Resource Availability area is not significant (0.75%), reflecting the current high level of availability of the materials and chemical products that comprise the battery components (Tabelin et al. 2021) and the normalization and weighting factors of the ReCiPe 2016 method for that area (Heijungs 2008).

The endpoint approach trends, outlined above, are in accordance with those obtained by other authors such as Shu et al. (2021), who carried out an LCA study of a 28.20-kWh LiFePO_4_ battery based on the ReCiPe 2016 method, obtaining endpoint values of 44.5 kPt, 0.05 kPt and 4.90·10^−4^ kPt (89.54%, 9.34% and 1.12%, respectively) for the Human Health, Ecosystem Quality and Resource Availability areas, respectively. However, their results differ significantly from those obtained in the present study (123, 7.08 and 0.97 mPt), mainly due to the significant difference in the FU considered in the two types of batteries (FU of 28.2 kWh which is 3775.10 times our FU of 7.47 Wh). Thus, if a scale change were made, this study would have obtained an impact of 118, 12.3 and 0.13 mPt. The slight variations observed are caused by using different environmental databases between studies (ELCD vs. Ecoinvent 3.5).

### Detailed consumption analysis

**Fig. 7:**
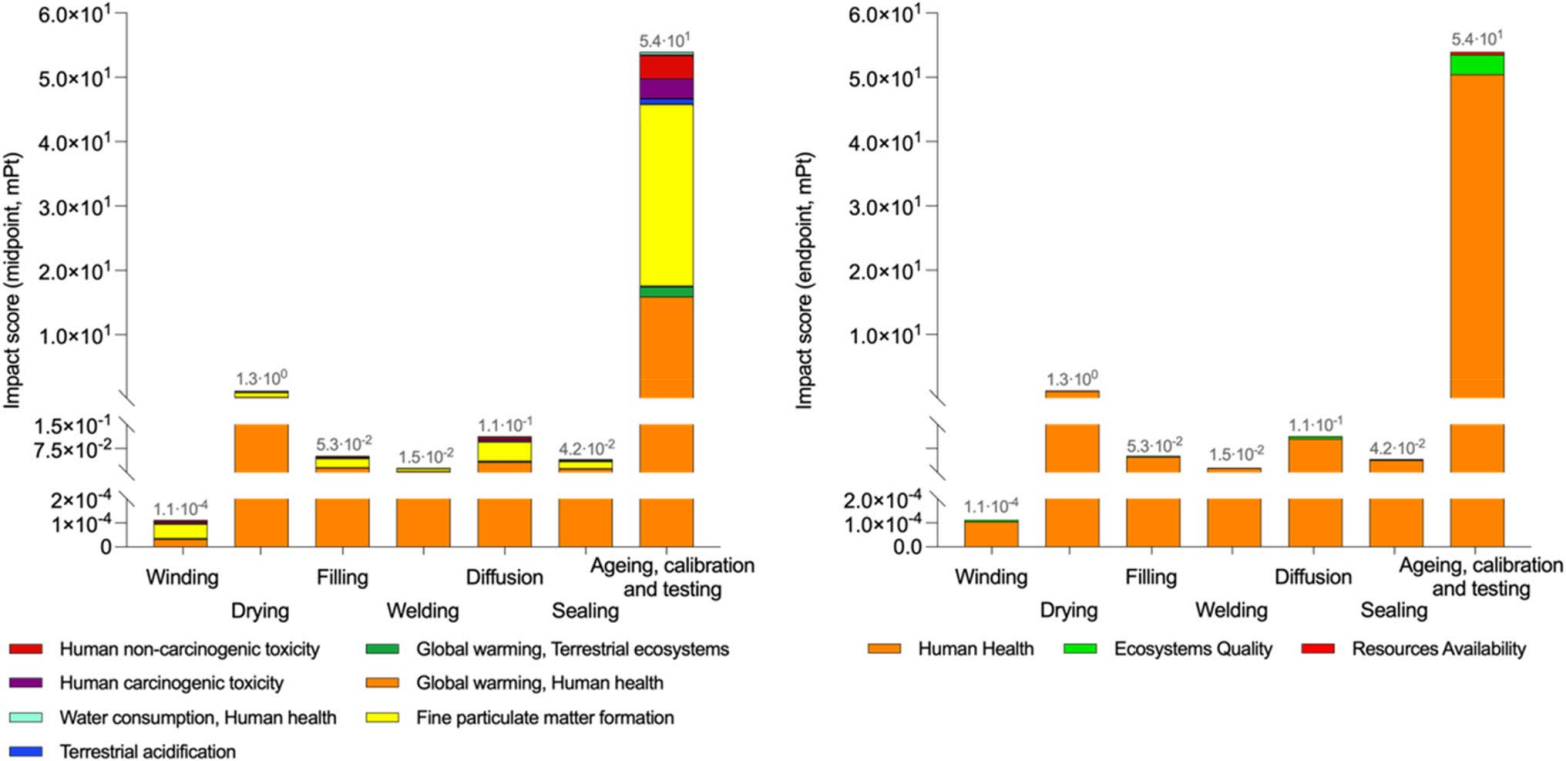
Impacts associated with the consumption of the cell battery’s production process (CBPP)

Figure 7 shows the detailed results for the environmental impact associated with the Li-ion 18,650 battery’s CBPP (in both the midpoint and the endpoint approaches). One observes that the ageing, calibration and testing process — P7 — has the highest environmental penalty with values of 53.97 mPt (97.21% of the CBPP total impact and 41.20% of the impact associated with the single-cell battery), of which 93.5% impact the area of Human Health, 5.7% that of Ecosystem Quality and 0.8% that of Resource Availability. This is attributable to the fact that this is a purely energetic process, dependent on the selected energy mix and whose penalising agents correspond to the damage pathways of the Human Health area (Steinmann et al. 2017). Also, the impact categories with the highest environmental penalty are those of fine particulate matter formation (28.01 mPt), global warming human health (15.92 mPt), human non-carcinogenic toxicity (3.18 mPt), human carcinogenic toxicity (2.85 mPt) and global warming terrestrial ecosystems (1.59 mPt). This is closely related to the SO_2_ and PM_2.5_ emissions derived from the energy flows of the Spanish energy mix, which have already been described in previous sections of this study. The following process with the highest environmental penalty is P2, but with impact values 40.88 times lower. This is due to the aforementioned use of economies of scale to minimize energy impact associated with FU. Finally, the process with least environmental penalty is P1 (1.12·10^−4^ mPt), as its energy demand is significantly lower (82,274.4 times lower) than the average of the rest of the energy processes.

### Uncertainty and sensitivity analysis

On the one hand, uncertainty is inherent to LCA due to its subjective character because of the particularities of each case study which are attributable to numerous assumptions and simplifications of the environmental impact assessment model. Nevertheless, using the ReCiPe method and the Ecoinvent 3.5 database which are well known and have been thoroughly tested and validated by the scientific community minimizes the uncertainty of our work.

On the other hand, this study included an exhaustive sensitivity analysis of the different possible locations of the battery production factory and of the supply of the stack’s raw materials and components. This will allow us to understand how these assumptions influence our results, i.e. in how the distance parameter affects the final results of the environmental impact of the battery. Table 7 lists the study case alternatives proposed for the sensitivity analysis.

**Table 7:** Case studies included in the sensitivity analysis

Case study	Sub-case	Description
C_0_	-	The baseline case study is defined in the “Material and methods”
C_I_	C_I–I_	5000 km supply distance for raw material increment
	C_I–II_	10,000 km supply distance for raw material increment
	C_I–III_	15,000 km supply distance for raw material increment
C_II_	C_II–I_	11,400-km distance decrement for the supply of electrodes and casing
	C_II–II_	6500-km distance decrement for the supply of electrodes and casing
	C_II–III_	3500-km distance increment for the supply of electrodes and casing

**Fig. 8:**
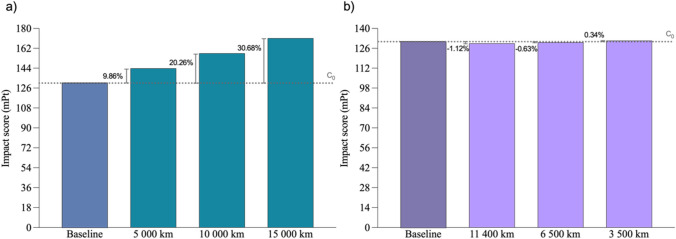
Sensitivity analysis of the distance parameter

Figure 8 shows the results obtained from the distance parameter sensitivity analysis for modifications related to the raw material supplies (C_I_) and to the suppliers’ locations (C_II_), as described by the case study in Table 7.

Analysing the raw material sourcing distance parameter, one observes that for the mean variations of the LCA parameter input of 933.5% (C_I–I_), 1766.67% (C_I–II_) and 2600.00% (C_I–III_), there were variations in the output of 9.86%, 20.26% and 30.68%, respectively. For a variation in the other input parameter to the model about the distance to suppliers, a marginal variation in the output parameter is obtained. Specifically, for variations in the input of the LCA parameter of 0.9% (C_II–I_), 43.48% (C_II–II_) and 130.4% (C_II–III_), there are variations in the output of 1.12%, 0.63% and 0.34%, respectively. So, for disproportionate variations in the distance parameter as an input of our model, relatively controlled variations are obtained in the output, which demonstrates the robustness of our model under changes in assumptions (Lo Piano and Benini 2022).

A final remark to confirm the validity of the insights of this investigation should be highlighted beyond robustness. These findings are taken from a well-established method widely checked by the scientific community, using a dual midpoint-endpoint approach typical of the ReCiPe 2016 method and a *“*cradle-to-gate*”* life cycle scenario. This duality broadens the scope of the results by offering two LCA perspectives. The “cradle-to-gate” scenario is one of the most established and least uncertain life cycle scenarios because, despite having a less detailed time frame than the “cradle-to-grave” scenario, it eliminates the uncertainties of the use and disposal stages (Scrucca et al. 2020). Notably, the ReCiPe 2016 method is very versatile, being applicable to different scenarios. For example, different battery component recycling processes can be compared to quantify the reduction of the environmental impact of each strategy (Wang et al. 2021). Furthermore, ReCiPe can be used to compare different types of lithium-ion batteries (Zhao and You 2019) or to compare different production processes of newly emerging materials (Schrijvers et al. 2016; Sazdovski et al. 2021).

## Conclusions

The comprehensive LCA of a 7.47-Wh 18,650 LiFePO_4_ cylindrical cell battery has allowed us to build an in-depth understanding about the environmental impact of all the components and processes associated with the production of a unit.

The most unfavourable source of environmental impact came from the consumptions associated with the CBPP, scoring highest in 16 of 22 midpoint impact normalized categories and in the three areas of damage — Human Health, Ecosystem Quality and Resource Availability. Furthermore, the disaggregated analysis showed that the ageing, calibration and testing process (P7) accounted for 97.21% of the total impact associated with consumptions and 41.20% of the impact associated with the battery’s full life cycle.

Moreover, the electrodes — anode and cathode — were the least sustainable, representing 71.82% of the environmental penalization of the components and 41.36% of the battery’s full life cycle. The separator was the least unfavourable component, making up 0.08% of the environmental penalty of the components and 0.05% of the battery’s full life cycle.

The current research verifies the importance of implementing the environmental dimension in the life cycle of Li-ion batteries to reduce their environmental impact. The results offer an overall and detailed vision of the environmental impacts associated with the different components and consumptions of the 18,650 cylindrical cell LiFePO_4_ battery, allowing producers of this battery to identify the critical points and the alternative solutions that can guarantee the Sustainable Development Goals.

Among the limitations of the study is the globalization of the weighting and normalization factors applied in the ReCiPe 2016 method, since this does not permit the analysis carried out to be adapted to all context environments, limiting the precision of the results obtained. In addition, any adaptation of the study for use in another country could lead to slight variations in the environmental impact results obtained due to modifications in the productive processes and to the country’s energy matrix or mix (Kisel et al. 2016), with the Spanish energy matrix being the one used in this study. However, the uncertainty and sensitivity of this research were minimized through different decisions, which guaranteed the robustness of the results.

Future research should focus on identifying and assessing recycled materials that can be incorporated into Li-ion batteries to minimize their environmental impact. To this end, an exhaustive analysis of different recycled materials potentially suitable from a technical point of view to replace elements of great environmental penalty such as electrodes should be carried out, subsequently subjecting them to a multidimensional evaluation process with which to classify, on the basis of environmental and techno-economic variables, the different solutions proposed. In addition, it would be interesting to analyse the relationship between the energy efficiency of the batteries and their LCA, evaluating the increase of environmental impact associated with the increase in power and energy density. For this purpose, a multivariate analysis considering representative parameters of energy consumption and power and energy density would have to be developed. The technique based on the Pareto front could be a very interesting tool to carry out this future research (Ghosh et al. 2023).

## Data Availability

The datasets used and/or analysed during the current study are available from the corresponding author upon reasonable request.

## Abbreviations

ADP: Abiotic depletion
AF: Acidification
CBPP: Cell battery’s production process
DALY: Disability-adjusted life years
DoD: Depth of discharge
FE: Freshwater eutrophication
FEC: Freshwater ecotoxicity
FPMF: Fine particulate matter formation
FRS: Fossil resource scarcity
FU: Functional unit
GHG: Greenhouse gases
GW: Global warming
GWFE: Global warming, freshwater ecosystems
GWHH: Global warming, human health
GWTE: Global warming, terrestrial ecosystems
HCT: Human carcinogenic toxicity
HnCT: Human non-carcinogenic toxicity
HT: Human toxicity
IR: Ionising radiation
LCA: Life cycle assessment
LCI: Life cycle inventory
Li-ion: Lithium-ion
LU: Land use
ME: Marine eutrophication
MEC: Marine ecotoxicity
MM: Metals and minerals
MRS: Mineral resource scarcity
NCM: Lithium, nickel, cobalt, manganese oxide
NCA: Lithium, nickel, cobalt, aluminium oxide
NOx: Nitrogen oxides
OFHH: Ozone formation, human health
OFTE: Ozone formation, terrestrial ecosystems
PM_10_: Particulate matter (10 µm)
PVDF: Polyvinylidene fluoride
SODP: Stratospheric ozone depletion
SO_x_: Sulphur oxides
TA: Terrestrial acidification
TE: Terrestrial ecotoxicity
US$: US dollar
WCAE: Water consumption, aquatic ecosystems
WCHH: Water consumption, human health
WCTE: Water consumption, terrestrial ecosystems
